# Antiplasmodial Properties and Cytotoxicity of Endophytic Fungi from *Symphonia globulifera* (Clusiaceae)

**DOI:** 10.3390/jof4020070

**Published:** 2018-06-12

**Authors:** Joël E. T. Ateba, Rufin M. K. Toghueo, Angelbert F. Awantu, Brice M. Mba’ning, Sebastian Gohlke, Dinkar Sahal, Edson Rodrigues-Filho, Etienne Tsamo, Fabrice F. Boyom, Norbert Sewald, Bruno N. Lenta

**Affiliations:** 1 Department of Organic Chemistry, Faculty of Science, University of Yaoundé 1, P.O. Box 812, 237 Yaoundé, Cameroon; atebaterence@yahoo.fr (J.E.T.A.); etsamo@yahoo.fr (E.T.); 2 Department of Biochemistry, Faculty of Science, University of Yaoundé I, Cameroon; P.O. Box 812, 237 Yaoundé, Cameroon; toghueo.rufin@yahoo.fr (R.M.K.T.); fabrice.boyom@fulbrightmail.org (F.F.B.); 3 Department of Chemistry, Faculty of Science, University of Bamenda, P.O. Box 39, 237 Bambili, Cameroon; aawantu@gmail.com; 4 Department of Chemistry, Organic and Bioorganic Chemistry, Bielefeld University, P.O. Box 100131, D-33501 Bielefeld, Germany; sebastian.gohlke@uni-bielefeld.de (S.G.); norbert.sewald@uni-bielefeld.de (N.S.); 5 International Centre for Genetic Engineering and Biotechnology, Aruna Asaf Ali Marg, New Delhi 110067, India; dsahal@gmail.com; 6 Departamento de Química-Universidade Federal de São Carlos-CP 676, São Carlos-SP 13565-905, Brazil; edinho@pq.cnpq.br; 7 Department of Chemistry, Higher Teacher Training College, University of Yaoundé 1, P.O. Box 47, 237 Yaoundé, Cameroon

**Keywords:** malaria, chloroquine-resistant *Plasmodium falciparum*, *Symphonia globulifera*, endophytic fungi, antiplasmodial IC_50_, selectivity index

## Abstract

There is continuing need for new and improved drugs to tackle malaria, which remains a major public health problem, especially in tropical and subtropical regions of the world. Natural products represent credible sources of new antiplasmodial agents for antimalarial drug development. Endophytes that widely colonize healthy tissues of plants have been shown to synthesize a great variety of secondary metabolites that might possess antiplasmodial benefits. The present study was carried out to evaluate the antiplasmodial potential of extracts from endophytic fungi isolated from *Symphonia globulifera* against a chloroquine-resistant strain of *Plasmodium falciparum (PfINDO*). Sixty-one fungal isolates with infection frequency of 67.77% were obtained from the bark of *S. globulifera*. Twelve selected isolates were classified into six different genera including *Fusarium*, *Paecilomyces*, *Penicillium*, *Aspergillus*, *Mucor,* and *Bipolaris*. Extracts from the 12 isolates were tested against *PfINDO*, and nine showed good activity (IC_50_ < 10 μg·mL^−1^) with three fungi including *Paecilomyces lilacinus* (IC_50_ = 0.44 μg·mL^−1^), *Penicillium janthinellum* (IC_50_ = 0.2 μg·mL^−1^), and *Paecilomyces* sp. (IC_50_ = 0.55 μg·mL^−1^) showing the highest promise. These three isolates were found to be less cytotoxic against the HEK293T cell line with selectivity indices ranging from 24.52 to 70.56. Results from this study indicate that endophytic fungi from *Symphonia globulifera* are promising sources of hit compounds that might be further investigated as novel drugs against malaria. The chemical investigation of active extracts is ongoing.

## 1. Introduction

Malaria remains a major cause of morbidity and mortality with more than 3.3 billion people living worldwide in countries with ongoing transmission at risk [1]. In 2016, 91 countries reported a total of 216 million cases of malaria and 445,000 deaths. The most affected populations were located in tropical and subtropical regions of the world, particularly in sub-Saharan Africa and Southeast Asia, where almost 80% of malaria cases are caused by *Plasmodium falciparum* [1]. The widespread appearance of drug-resistant malaria parasites, even to newly developed second and third generation therapeutics such as artemisinin and its derivatives, makes the development of novel antimalarial drug treatments all the more urgent [2].

Natural products remain a consistent source of antimalarial drug leads, with the best examples being quinine and artemisinin that have acted as gifts of medicinal plants for victims of malaria. Therefore, investigating natural resources for antiplasmodial drug discovery continues to be one of the best scientific approaches that can lead to the identification of novel lead compounds against malaria [3]. Moreover, in comparison to other natural sources like plants, microorganisms are highly diverse but narrowly explored. Indeed, microbes often coexist with plants and animals making it difficult to assess if the real hosts of the promising metabolites identified are from higher organisms themselves or the tiny microbes residing in them. However, studies based on estimation of microbial populations have revealed that only about 1% of bacteria and 5% of fungi have been characterized and the rest remain unexplored for their potential to offer novel drugs against diverse diseases [4]. One of these groups being explored in recent times for their drug discovery potential is endophytic fungi from medicinal plants.

In fact, during the last 20 years it has been observed that much of the wealth of microbial biodiversity with novel biochemistry and secondary metabolite production resides in endophytic association with plant tissues [5]. Interest in such microorganisms, termed endophytes, increased immensely with the discovery of an endophytic fungus, from *Taxus brevifolia*, producing the billion dollar anti-cancer drug taxol [6]. Numerous bioactive molecules have been isolated from endophytic fungi since this groundbreaking discovery [7,8]. Endophytes are metabolically more active than their free counterparts due to their specific functions in nature and activation of various metabolic pathways needed to survive in the host tissues [4,9,10]. Therefore, investigation of endophytic fungi from medicinal plants used to treat malaria can lead to new antimalarial drug discovery.

*Symphonia globulifera* L. f., an evergreen tree of the Clusiaceae family, is widely distributed in Central and South America, and in tropical Africa, from Sierra Leone to Uganda, Zambia, and Angola. In traditional medicine, a decoction of leaves from this plant is used to manage malaria and several other diseases, including diabetes and skin diseases [11]. In addition, decoctions and extracts of bark are used as diuretics and antiparasitics, and to treat river blindness, chest complaints, cough in children, gonorrhea, scabies, intestinal worms, and prehepatic jaundice [12,13]. Therefore, the present study was designed to investigate for the first time antiplasmodial activity of endophytic fungi isolated from the bark of *S. globulifera.*

## 2. Materials and Methods

### 2.1. Collection of Plant Material

Healthy and mature stem bark of *Symphonia globulifera* L. f. was collected at Nomayos in the Centre Region of Cameroon in January 2015, and was identified at the National Herbarium of Cameroon, where a voucher specimen was deposited under the number 32192/HNC. Plant material was directly brought to the laboratory in sterile bags and processed within a few hours after sampling.

### 2.2. Isolation of Endophytic Fungi

Stem bark was rinsed with tap water and cut into small pieces, approximately 5 mm length. Sterilization was assessed by means of a 5 min rinse with ethanol, followed by treatment with a 1% active chlorine solution for 15 min, 2 min in ethanol, and a final rinse in sterile water [14].

After surface sterilization, six plates of potato dextrose agar (PDA) supplemented with chloramphenicol (200 mg·L^−1^), each containing 15 pieces of individual plant material, were prepared and kept in the dark at room temperature (22–26 °C). Fragments of mycelium emerging from plant pieces were transferred to new PDA plates without chloramphenicol to obtain pure cultures for identification. To assess whether disinfection methods were effective in eliminating surface fungi, imprints of treated fragments were made by pressing them against the surface of fresh PDA plates that were incubated without plant parts. These plates were checked for fungi emerging from the prints [14].

### 2.3. Identification of Endophytic Fungi

Fungal cultures were maintained at room temperature (i.e., 22–26 °C) under a natural photoperiod for 10–21 days and then examined visually for macroscopic (i.e., morphology, size, and coloration of the mycelium and agar media) and microscopic (i.e., presence of spores or other reproductive structures) characteristics. Colonies were analyzed with respect to their average diameter, sporulation, and the size and coloration of the conidia. Isolates having similar culture characteristics were grouped into morphotypes. From morphotype groups, only one isolate was processed for further identification.

Identification of endophytic isolates representative of each morphotype was based on the nucleotide sequence of the ITS1-5.8S rRNA-ITS2 region. DNA was extracted from samples of mycelium from the fungal cultures using a commercial kit (RedExtract-N-Amp Plant PCR, Sigma Aldrich, USA). The ITS1-5.8S rRNA-ITS2 region was amplified in a polymerase chain reaction (PCR) using primers ITS4 and ITS5 and the protocol described by White et al. [15]. Amplicons were purified by filtration (MSB Spin PCRapace, Invitek, Germany) and sequenced.

The FASTA algorithm was used to find sequences similar to those obtained from fungal isolates. The criteria for identification of isolates were based on the similarity of their sequences to those of reliable reference isolates included in public nucleotide databases. To visualize the diverse fungal taxa identified sequences, a dendrogram was made with the ITS1-5.8S rRNA-ITS2 nucleotide sequences of the isolates and those of reference strains deposited in CBS (Centraalbureau voor Schimmelcultures), ATCC (American Type Culture Collection), or other fungal collections, as well as some used in published works of fungal taxonomy. Sequences were aligned using the Clustal X 2.1, and the dendrogram was made with MEGA 6.06 software using the neighbor-joining method with Kimura 2-parameter distances. Groups of sequences at close proximity within the same branch of the dendrogram were individually aligned with Clustal X 2.1 to determine their percentage of similarity. Sequences with a similarity greater than 99% were considered to belong to the same species [14].

### 2.4. Fermentation and Extraction

Each fungus was cultivated on 2 kg of rice by placing agar blocks of actively growing pure culture (3 mm in diameter) in a 1000 mL Erlenmeyer flask. Each flask was incubated at 25 ± 2 °C for 30 days. After incubation, moldy rice was macerated with ethyl acetate and filtered through three layers of muslin cloth. The organic phase was collected and the solvent was then removed by evaporation under reduced pressure at 40 °C using a rotary vacuum evaporator. The dry solid residues were examined for their antiplasmodial activity and cytotoxicity.

### 2.5. Biological Assays

#### 2.5.1. In Vitro Cultivation of *Plasmodium falciparum*

A chloroquine-resistant *PfINDO* strain of *P. falciparum* was maintained in a continuous culture following the method of Trager & Jensen, 1976 [16] with minor modifications. Cultures were maintained in fresh O positive human erythrocytes suspended at 4% (*v/v*) haematocrit in complete medium (16.2 g·L^−1^ RPMI 1640 (Sigma) containing 25 mM HEPES, 11.11 mM glucose, 0.2% sodium bicarbonate (Sigma), 0.5% Albumax I (Gibco), 45 mg·L^−1^ hypoxanthine (Sigma), and 50 mg·L^−1^ gentamicin (Gibco)) and incubated at 37 °C in a gas mixture consisting of 5% O_2_, 5% CO_2_, and 90% N_2_. The spent medium was replaced with fresh complete medium every day to propagate the culture. Giemsa-stained blood smears were examined microscopically to monitor cell cycle transitions and parasitemia.

#### 2.5.2. Preparation of Stock Solution of Chloroquine and Fungal Extracts

Stock solutions of fungal extracts were prepared in Dimethyl sulfoxide (DMSO) at 25 mg·mL^−1^ and that of chloroquine phosphate (CQ) (Sigma) was prepared at 1 mM in Milli-Q grade water. The required drug concentrations were achieved by diluting the stocks with incomplete RPMI (Roswell Park Memorial Institute) 1640 medium. The solutions of drugs and extracts were placed in 96-well flat-bottom tissue culture grade plates (Corning). For each extract, the concentrations (μg·mL^−1^) tested were 0.195, 0.39, 0.78, 1.562, 3.125, 6.25, 12.5, 25, 50, and 100.

#### 2.5.3. In Vitro Antiplasmodial Assay

Before each experiment, synchronized ring stage parasites were obtained by 5% (*w/v*) sorbitol treatment [17]. It is important to note that use of synchronized cultures over mixed-stage cultures can enable the test molecules to interact with all three stages (i.e., ring, trophozoite, and schizont) of the 48 h long life cycle of *P. falciparum* in culture. Moreover, starting the experiment with synchronized ring stage culture provides the distinct advantage of observing growth inhibitory effects without a rise in parasitemia during the ring-trophozoite-schizont transitions.

For drug screening, the SYBR green I based fluorescence assay was used [18]. The ability of SYBR green to give strong fluorescence only in the presence of DNA forms the basis for its use to assess cell proliferation. The absence of a nucleus in human red blood cells where the malaria parasite proliferates allows the use of SYBR green for the specific monitoring of the growth of malarial parasite.

Sorbitol-synchronized ring stage parasites (haematocrit: 2%, parasitemia: 1%, 96 µL) under normal culture conditions were incubated in the presence or absence of increasing concentrations of the extracts. Four µL CQ (1 mM) was used as a positive control and 0.4% DMSO (*v/v*), which was found to be non-toxic to the parasite, was used as vehicle control. After 48 h of incubation, 100 µL of SYBR Green I buffer {0.2 µL of 10,000 × SYBR Green I (Invitrogen) per mL of lysis buffer (Tris (20 mM; pH 7.5), EDTA (5 mM), saponin (0.008%; *w/v*), and Triton X-100 (0.08%; *v/v*)} was added to each well, mixed twice gently with multi-channel pipette and incubated in the dark at 37 °C for 1 h. Fluorescence was measured using a Victor fluorescence multi-well plate reader (Perkin Elmer) with excitation and emission at 485 and 530 nm, respectively. Fluorescence counts for CQ representing zero growth were deducted from counts in each well. A dose–response curve was constructed by plotting fluorescence counts against the drug concentration and IC_50_ (dose of a drug required to retard the growth of a cell population by 50%) was determined using IC Estimator-version 1.2 (http://www.antimalarial-icestimator.net/MethodIntro.htm). In this experiment, no drug (control) corresponds to 100% growth while 40 µM chloroquine (sufficient to cause total arrest of growth) corresponds to 0% growth. Giemsa-stained smears of extract-treated parasite cultures were visualized microscopically to validate the results from fluorescence-based assay. Experiments were done in triplicate and means with standard deviation were calculated. Extracts were classified according to criteria of the antiplasmodial activity based upon good (IC_50_ < 10 μg·mL^−1^), moderate (IC_50_ > 10 to < 25 μg·mL^−1^), and inactive (IC_50_ > 25 μg·mL^−1^) activity as proposed by Bagavan et al. [19].

#### 2.5.4. Cytotoxicity Assay

The cytotoxic effects of the most potent crude extracts were determined by a functional assay [20] using the human embryonic kidney HEK239T cells cultured in complete medium containing 16.2 g·L^−1^ DMEM, 10% fetal bovine serum, 0.2% sodium bicarbonate (*w*/*v*) (Sigma), and 50 μg·mL^−1^ gentamycin. Cells (5 × 10^3^ cells/200 µL/well) were seeded into 96-well flat-bottom tissue culture plates in complete medium. After 24 h of cells seeding, test extracts were added and cells incubated for 48 h in a humidified atmosphere at 37 °C and 5% CO_2_. DMSO (as positive inhibitor) was added at 10% *v*/*v*. Twenty microliters of a stock solution of MTT [3-(4,5-dimethylthiazol-2-yl)-2,5-diphenyltetrazolium bromide] (5 mg·mL^−1^ in 1× phosphate buffered saline) was added to each well, gently mixed, and incubated for another 4 h. The supernatant was thereafter removed and 100 µL DMSO (quench agent) was added to the cell pellet. Formazan formation was measured using a microtiter plate reader (VersaMax tunable multiwell plate reader) at 570 nm. The 50% cytotoxic concentration (CC_50_) of drugs was determined by analysis of dose–response curves. Selectivity index (CC_50_/IC_50_) was calculated for each extract (tested in triplicate).

## 3. Results

### 3.1. *Isolation and Identification of Fungi*

**Figure 1:**
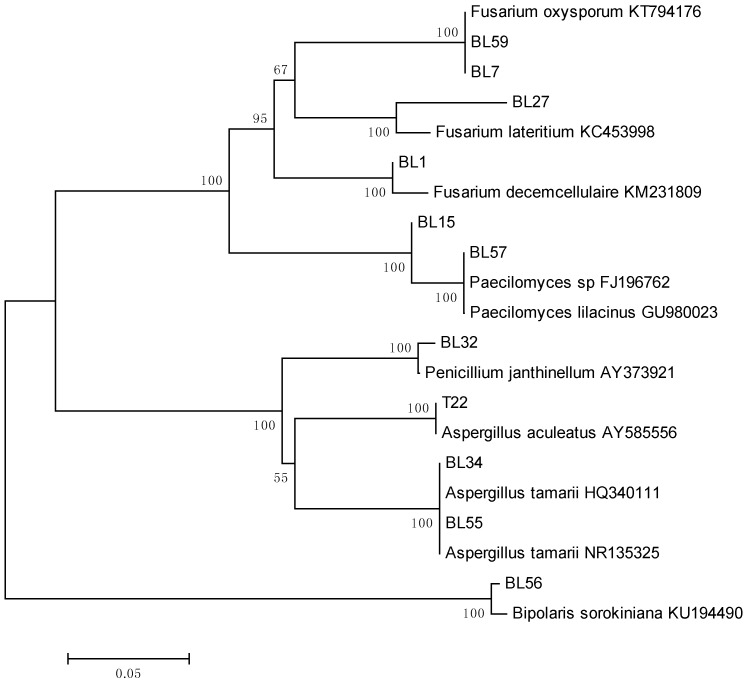
Classification of fungal endophytes isolated from *Symphonia globulifera* based on a neighbor-joining phylogenetic tree made with ITS1-5.8S rDNA-ITS2 nucleotide sequences. Taxa with accession numbers correspond to reference strains. Numbers at branch nodes are bootstrap values based on 500 replications.

From a total of 90 fragments of the stem bark of *Symphonia globulifera*, 61 isolates were obtained with an infection frequency of 67.77%. They were subsequently grouped into 14 morphotypes and one isolate from each morphotype was identified by sequence analysis of the Internal Transcribed Spacer (ITS) region. Twelve analyzed isolates were classified into six different genera including, *Fusarium*, *Paecilomyces*, *Penicillium*, *Aspergillus*, *Mucor,* and *Bipolaris* (Table 1; Figure 1). The most representative genera were *Fusarium* and *Penicillium* (Table 1).

### 3.2. In Vitro Antiplasmodial Activity of Endophytic Extracts

Twelve endophytic fungi were cultured in a rice medium at 25 ± 2 °C for 30 days and subsequently extracted using ethyl acetate as a solvent. The extraction yields ranged from 6.30 g to 21.70 g with *A. tamarri* producing the higher yield.

The IC_50_ of the tested extracts against the chloroquine-resistant *Plasmodium falciparum* INDO (*PfINDO*) strain ranged from 0.2 to >100 μg·mL^−1^ (Table 2). Out of the 12 tested extracts, nine showed good activity (IC_50_ < 10 μg·mL^−1^). Extracts from *Paecilomyces lilacinus, Penicillium janthinellum*, and *Paecilomyces* sp. exerted highly potent activities with IC_50_ < 1 μg/mL. Extracts from *Mucor falcatus* and *Aspergillus aculeatus* showed moderate potency with IC_50_ between 10 and 25 μg·mL^−1^, while the extract from *Aspergillus tamarri* with IC_50_ > 100 μg·mL^−1^ was considered as inactive.

### 3.3. Cytotoxicity of Extracts

The cytotoxicity of the extracts was tested against HEK239T cells. As shown in Table 2, CC_50_ values (µg mL^−1^) ranged from 3.06 to 70.91 leading selectivity indices to lie between 0.5 and 70.56. *Penicillium* sp. (2). (SI = 0.69), *Bipolaris sorokiniana* (SI = 0.504), and *Penicilliun* sp. (1) (SI = 0.842) were found to be highly cytotoxic against HEK239T cells. Conversely, the most potent fungal extracts from *Paecilomyces lilacinus* (CC_50_ = 10.79 µg mL^−1^; SI = 24.52), *Penicillium janthinellum* (CC_50_ = 9.14 µg mL^−1^; SI = 45.7), and *Paecilomyces* sp. (CC_50_ = 38.88 µg mL^−1^; SI = 70.56) were less cytotoxic.

## 4. Discussion

Endophytic fungi have been claimed to be responsible for the medicinal properties of several medicinal plants [21]. Therefore, in this study we have explored the antiplasmodial potential of endophytic fungi associated with *Symphonia globulifera* used in several African countries for treatment of malaria. More specifically, towards discovery of new drugs against malaria, this study was done to perform antiplasmodial screening of endophytic fungi obtained from the bark of this plant.

A high prevalence of endophyte infection of 67.77% was found in bark fragments of *Symphonia globulifera*. This high level of endophyte colonization is corroborated by previous findings which indicated that endophyte prevalence in some tissues of Cameroonian medicinal plants is quite high [14]. Moreover, Arnold and Lutzoni [22] reported that the prevalence of endophyte species in plant tissues of tropical regions is greater than in plant species of temperate and boreal forests. The identification based on sequence analysis indicated that all of the analyzed endophytic isolates obtained from bark of *S. globulifera* belong to six different genera including *Fusarium*, *Paecilomyces*, *Penicillium*, *Aspergillus*, *Mucor,* and *Bipolaris*. All these genera have also been reported in previous endophyte surveys in different plant species [14,23,24,25,26].

Although only 12 endophyte isolates were screened in the present study, as many as nine of them produced antiplasmodial compounds [27,28]. The finding that as high as 75% gave good activity (IC_50_ < 10 μg·mL^−1^) with 33.3% (3) of this portion showing highly potent antiplasmodial activity (IC_50_ < 1 μg·mL^−1^) suggests that deeper exploration of endophytic fungi may reveal a rich repertoire of antiplasmodial molecules. Previous studies reported that endophyte fungi of *Penicillium* and *Paecilomyces* species are important sources of antiplasmodial secondary metabolites [28]. In fact, β-Resorcyclic acid lactones, cyclodepsipeptides, and tropane derivatives with potent antiplasmodial activities have earlier been isolated from *Paecilomyces* [29,30,31] and *Penicillium* [27] species.

Our findings have indicated that endophytic fungi belonging to the genera *Fusarium*, *Paecilomyces*, *Penicillium*, and *Bipolaris* isolated from this plant species should be studied intensely for novel antiplasmodial compounds. Therefore, further studies on the antiplasmodial activity guided isolation and characterization of active metabolites produced by promising fungi necessary for the discovery of new drugs against malaria are ongoing.

## 5. Conclusions

This study is the first report on the antiplasmodial potential of endophytic fungi from the bark of *Symphonia globulifera*. In this investigation, 9 out of 12 fungi showed promising antiplasmodial potency with *Paecilomyces lilacinus*, *Penicillium janthinellum*, and *Paecilomyces* sp. being the most promising*.* These results obtained in this study highlight endophytic fungi from *S. globulifera* as a promising source of novel lead antimalarial compounds.

## Figures and Tables

**Table 1:** Identification of endophyte isolates and percentage homology of sequences.

Endophyte Isolates	**% Sequence Homology**	**Organism with the Highest Sequence Identity, GenBank Acc. No.**
*Fusarium decemcellulaire*	98	*Fusarium decemcellulaire* KM231809
*Paecilomyces* sp.	100	*Paecilomyces* sp. FJ196762
*Fusarium oxysporum*	100	*Fusarium oxysporum* KT794176
*Paecilomyces lilacinus*	100	*Paecilomyces lilacinus* GU980023
*Penicillium janthinellum*	99	*Penicillium janthinellum* AY373921
*Aspergillus tamarri*	100	*Aspergillus tamarri* HQ340111
*Penicilium* sp. (1)	99	*Penicillium* sp. JN021538
*Bipolaris sorokiniana*	94	*Bipolaris sorokiniana* KU194490
*Fusarium lateritium*	94	*Fusarium lateritium* KC453998
*Penicillium* sp. (2)	98	*Penicillium* sp. JN021538
*Aspergillus aculeatus*	100	*Aspergillus aculeatus* AY585556
*Mucor falcatus*	89	*Mucor falcatus* NR103647

**Table 2:** Antiplasmodial and cytotoxic activities of extracts of endophytic fungi from the bark of *Symphonia globulifera*.

Sample	Dry Mass of Extract (g)	IC_50_ against *PfINDO* Strain (μg·mL^−1^ ± SD)	CC_50_ against *HEK239T* Cells (μg·mL^−1^ ± SD)	SI (CC_50_/IC_50_)
*Fusarium decemcellulaire*	10.81	2.19 ± 0.07	70.91 ± 0.18	32.37
*Paecilomyces* sp.	14.66	0.55 ± 0.01	38.81 ± 0.19	70.56
*Fusarium oxysporum*	8.23	1.70 ± 0.22	18.24 ± 0.11	10.729
*Paecilomyces lilacinus*	6.20	0.44 ± 0.03	10.79 ± 0.2	24.52
*Penicillium janthinellum*	6.30	0.20 ± 0.01	9.14 ± 0.12	45.7
*Aspergillus tamarri*	21.70	>100	***	NA
*Penicilium* sp.	14.57	3.63 ± 0.02	3.06 ± 0.36	0.842
*Bipolaris sorokiniana*	9.89	6.10 ± 0.37	3.08 ± 0.19	0.504
*Fusarium lateritium*	11.67	6.61± 0.01	2.82± 0.23	0.426
*Penicillium* sp.(2)	16.32	9.08 ± 0.13	6.28 ± 1.23	0.69
*Aspergillus aculeatus*	8.74	22.08 ± 0.43	***	NA
*Mucor falcatus*	9.22	17.51 ± 0.19	***	NA
*Chloroquine*	NA	*400	***	NA

Data are presented as mean values ± standard deviation of triplicate experiments; SD: standard deviation; ***: not tested; NA: not applicable. SI: Selectivity index (ratio of CC_50_ to IC_50_); * IC_50_ of chloroquine given in nM.
